# How to consider the indication of leadless pacemaker versus conventional transvenous pacemaker following their comparison in cost‐effectiveness

**DOI:** 10.1002/joa3.13198

**Published:** 2024-12-06

**Authors:** Naoya Kataoka, Teruhiko Imamura

**Affiliations:** ^1^ Second Department of Internal Medicine University of Toyama Toyama Japan

To Editor,

Micra VR, a single‐chamber, leadless trans‐catheter pacing system, represents a significant advancement in pacemaker technology. By eliminating the need for traditional pacemaker leads and subcutaneous pockets, it substantially reduces the risk of infection—a primary complication associated with conventional transvenous pacemakers (TVPM). In a comprehensive study conducted within a large‐scale Australian healthcare system, Makino and colleagues assessed the cost‐effectiveness of Micra VR in comparison with TVPM.^1^ Their findings revealed a higher incidence of procedure‐related complications in the Micra VR group; however, the costs associated with device‐related infections and re‐implantations of devices were notably higher in the TVPM group. Consequently, Micra VR demonstrated superior cost‐effectiveness in managing patients with symptomatic bradycardia. Several concerns have been raised.

Beyond infection reduction, Micra VR offers additional advantages, such as preserving systemic venous access critical for patients requiring hemodialysis, as well as feasibility for patients with superior vena cava obstruction or those with replaced tricuspid valves.^2^, ^3^ In this study, the outcomes assessed included infection, infectious endocarditis, device revisions, repeated lead implantations, repeated device implantations, and device retrievals.^1^ The cost‐effectiveness of Micra VR may be even more pronounced when considering other TVPM‐associated adverse events, such as the need for repeated shunt creation due to TVPM‐related vascular obstruction, bradycardia‐induced exacerbation of heart failure from unsuccessful TVPM implantation, and surgical implantation of epicardial leads following tricuspid valve replacement.

Leadless pacemakers are typically preferred for elderly patients, likely due to the limitations of Micra AV in achieving optimal AV synchronization.^4^ Nonetheless, in this study, Micra VR was associated with a higher quality of life than TVPM, potentially attributable to the absence of a subcutaneous pocket. Given this improvement in quality of life, Micra VR could, in fact, be considered a viable option for younger patients as well. How do the authors evaluate the clinical indications for Micra VR in comparison with TVPM in the contemporary era?

## FUNDING INFORMATION

None.

## CONFLICT OF INTEREST STATEMENT

The authors declare no conflicts of interest.

## Data Availability

The manuscript does not include any original data.

## References

[1] Makino K , Mudge M , Hill M , Zaunmayr C , Tilden D . Cost‐effectiveness of Micra™ VR leadless pacemaker in patients with bradycardia and atrial fibrillation in Australia. Journal of Arrhythmia. 2024;00:1–9. 10.1002/joa3.13145 PMC11632246 39669916

[2] El‐Chami MF , Clementy N , Garweg C , Omar R , Duray GZ , Gornick CC , et al. Leadless pacemaker implantation in hemodialysis patients: experience with the Micra transcatheter pacemaker. JACC Clin Electrophysiol. 2019;5:162–170. 30784685 10.1016/j.jacep.2018.12.008

[3] Okabe T , Afzal MR , Houmsse M , Makary MS , Elliot ED , Daoud EG , et al. Tine‐based leadless pacemaker: strategies for safe implantation in unconventional clinical scenarios. JACC Clin Electrophysiol. 2020;6:1318–1331. 33092762 10.1016/j.jacep.2020.08.021

[4] Garweg C , Chinitz JS , Marijon E , Haeberlin A , Winter S , Iacopino S , et al. A leadless ventricular pacemaker providing atrioventricular synchronous pacing in the real‐world setting: 12‐month results from the Micra AV post‐approval registry. Heart Rhythm. 2024;21:1939–1947. 38878939 10.1016/j.hrthm.2024.06.008

